# Supplementary material for: The Evolution and Characterization of the RNA Interference Pathways in Lophotrochozoa
Source: Genome Biol Evol. 2024 May 7;16(5):evae098. doi: 10.1093/gbe/evae098 (PMC11114477; doi:10.1093/gbe/evae098)
Supplement: evae098_Supplementary_Data [file evae098_supplementary_data.zip › Supplementary_materials.zip!/selected_miRNAs/NoGe_selected_miRNAs/novel_miRNA_families_pdf_files/NMRB01001012.1_Notospermus_geniculatus_strain_Ushimado_scaffold1012,_whole_genome_shotgun_sequence_23918.pdf]

| | | |
|---|---|---|
| Provisional ID | : | NMRB01001012.1_Notospermus_geniculatus_strain_Ushimado_scaffold1012,_whole_genome_shotgun_sequence_23918 |
| Score total | : | 481.9 |
| Score for star read(s) | : | 3.9 |
| Score for read counts | : | 477.3 |
| Score for mfe | : | 1.3 |
| Score for randfold | : | 0 |
| Score for cons. seed | : | -0.6 |
| Total read count | : | 948 |
| Mature read count | : | 921 |
| Loop read count | : | 0 |
| Star read count | : | 27 |

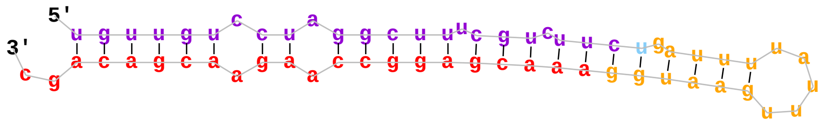


Genome coordinates: NMRB01001012.1 182228-182290

pre-miRNA sequence:
TGTTGTCCTAGGCTTTCGTCTTCTGTTGTCCTAGGCTTTCGTCTTCTGATTTTATTTGAATGGAAACGAGGCCAAGAACGACAGC

Mature sequence: AAACGAGGCCAAGAACGACAGC

BLAST results for the pre-miRNA sequence against the *Lineus longissimus* genome assembly:

| **Scaffold** | **Start** | **Stop** | **Identity** | **e-value** |
|---|---|---|---|---|
| OU343004.1 1 | 3392306 | 3392246 | 95.082 | 8.48e-20 |

```
Nge_novel_miRNA        TGTTGTCCTAGGCTTTCGTCTTCTGATTTTATTTGAATGGAAACGAGGCCAAGAACGACAG
Llo_genome             TGTTGTCCGAGGCTTTCGTCTTCTGATATTGTTTGAATGGAAACGAGGCCAAGAACGACAG
```
